# Association of PM_2.5_ concentration with health center outpatient visits for respiratory diseases of children under 5 years old in Lima, Peru

**DOI:** 10.1186/s12940-020-0564-5

**Published:** 2020-01-15

**Authors:** Jennifer Estefanía Davila Cordova, Vilma Tapia Aguirre, Vanessa Vasquez Apestegui, Luis Ordoñez Ibarguen, Bryan N. Vu, Kyle Steenland, Gustavo F. Gonzales Rengifo

**Affiliations:** 1 Faculty of Sciences and Philosophy, and Laboratory of Investigation and Development, Universidad Peruana Cayetano Heredia, Lima, Peru; 2 National Center for Epidemiology, Prevention and Control of Diseases, Minsa, Peru; 3 Department of Environmental Health, Rollins School of Public Health, Emory University, Atlanta, GA 30322 USA

**Keywords:** Health center outpatient visits, PM2.5, Respiratory diseases, Air pollution, Children

## Abstract

**Background:**

Lima is one of the more polluted cities in Latin America. High levels of PM_2.5_ have been shown to increase health center outpatient visits of respiratory diseases.

**Methods:**

Health center outpatient visits for children < 5 years for childhood respiratory disease (acute lower respiratory infections (ALRI), pneumonia and acute bronchiolitis/asthma) from 498 public clinics in Lima were available on a weekly basis from 2011 to 2015 from Peru’s Ministry of Health (MINSA). The association between the average weekly concentrations of PM_2.5_ was evaluated in relation to the number of weekly health center outpatient visits for children. Weekly PM_2.5_ values were estimated using a recently developed model that combined data observed from ground monitors, with data from space satellite and meteorology. Ground monitoring data came from 10 fixed stations of the Peruvian National Service of Meteorology and Hydrology (SENAMHI) and from 6 mobile stations located in San Juan de Miraflores by Johns Hopkins University. We conducted a time-series analysis using a negative binomial model.

**Results:**

We found a significant association between exposure to PM_2.5_ and all three types of respiratory diseases, across all age groups. For an interquartile increase in PM_2.5_, we found an increase of 6% for acute lower respiratory infections, an increase of 16–19% for pneumonia, and an increase of 10% for acute bronchiolitis / asthma.

**Conclusions:**

Higher emissions of environmental pollutants such as PM_2,5_ could be a trigger for the increase of health center outpatients visits for respiratory diseases (ALRI, pneumonia and asthma), which are themselves risk factors for mortality for children in Lima province, Peru.

## Background

A World Health Organization (WHO) report regarding global outdoor air pollution in 2014 noted that Lima, the capital of Peru, was one of more polluted cities in the Americas [1]. Average PM_2.5_ levels during 2014–2015 were 26 μg/m^3^ [2]. Ambient air pollution has been associated with respiratory diseases in children [3]. Environmental air pollution is one of the causes of mortality and morbidity, due to cardiovascular diseases, acute respiratory infections, pneumonias and acute bronchiolitis/asthma [4, 5]. Respiratory diseases are among the leading causes of death in the world [6].

According to the WHO, acute lower respiratory infections (ALRI) cause the death of 4.3 million children under 5 years old, which represents 30% of the total annual deaths of children in this age group [6]. Also ALRI are the leading cause of premature death in Peru, approximately 222 child deaths per year for every 100,000 live births [6].

PM_2.5_ are fine particles with aerodynamic diameter of 2.5 μm or less, which are emitted from a large variety of sources including automotive vehicles, industry, power generation and engine combustion [7, 8]. Some prior studies have found an association between PM_2.5_ and increased health center outpatient visits for respiratory diseases [9]. It has also been shown that PM_2.5_ levels have a greater effect on admission to outpatient health clinics due to respiratory problems than do PM_10_ levels [4]. Childhood respiratory diseases may be partially preventable with better control of environmental contaminants, such as PM_2.5_ [10].

We evaluated the association between the average weekly concentration of PM_2.5_, and the number of weekly health center outpatients visits for ALRI, pneumonia and acute bronchiolitis/asthma in children under 5 years old in public health centers in Lima province, Peru between 2011 and 2015.

## Methods

We conducted a time-series analysis, with data related to health center outpatient visits due to childhood respiratory diseases, including acute lower respiratory inflammatory, pneumonia, asthma. Children with respiratory diseases were divided according to respiratory diseases and age groups: ALRI (acute lower respiratory disease, J00-J11.1 ICD-10 (International Classification of Diseases 10th)) from 0 to 2 months, 2 to 11 months and 1 to 5 years; pneumonia (J12.0-J18.9 ICD-10) from 2 to 11 months and 1 to 4 years; and acute bronchiolitis/asthma (J21.0-J21.9 / J44.8–46.0 ICD-10)(acute bronchiolitis (J21) and asthma (J44.8-J46) are similar diseases and may have similar causes). Data were grouped by age, with categories varying slightly by disease category. The age and disease groupings are those used by Peru’s Ministry of Health (MINSA), and we received the data with these groupings.

Our study included all child outpatients who lived in Lima province, considering all the districts (43 districts) of the Lima province. In the Lima province, there are 498 MINSA health centers which receive outpatients. The number of health center outpatient visits was obtained for January to December of 2011–2015 (*n* = 3,099,438); cases were available only grouped by week, so weekly cases were our unit of analysis. The outcome data were obtained from the National Center for Epidemiology, Prevention and Disease Control, a part of the Peruvian Ministry of Health (MINSA).

PM_2.5_ values were estimated using a model that combined data observed from ground monitors, with space satellite and meteorological data, to estimate daily PM_2.5_ at a 1 km^2^ resolution. The ground monitoring was carried out at 10 fixed stations of the National Service of Meteorology and Hydrology of Peru (SENAMHI) and from 6 mobile stations located in San Juan de Miraflores, collected by Johns Hopkins University [7]. This model was a good predictor of the observed ground monitoring data (R-square = 0.70).

Daily PM_2.5_ estimates by district, weighted by population density, were averaged to get weekly means for each district where a patient lived. District of residence was available from health center records. The weekly mean temperature, weekly mean relative air humidity, seasons (summer, autumn, fall and spring), years, indicator variables for districts, all of which may act as confounders, were included as variables in the models; the meteorological data were obtained from SENAHMI [6]. The analysis includes 11,050 observations (52 weekly visits * 5 years * 43 districts), with a 1.2% loss of observations due to missing data.

The statistical analysis was first done via a negative binomial model (the Poisson model was over-dispersed) with week-long lags of 0 to 3 weeks. Goodness of fit was evaluated using the Akaike Information Criterion (AIC). The rate ratio for health center outpatient visits was estimated for an increase in the interquartile range (IQR) of PM_2.5_, the increase from the 25th to the 75th percentiles (which was 7.1 μg/m^3^ during the years considered). Also, we analyzed PM_2.5_ as a categorical variable in quintiles (Q): 1stQ: < 15.64 μg/m^3^; 2ndQ: 15.64–17.48 μg/m^3^; 3rdQ:17.49–19.71 μg/m^3^; 4thQ: 19.72–25.08 μg/m^3^ and 5thQ: 25.09–48.62 μg/m^3^.

The inclusion of a dummy variable for district in the model essentially stratified on district, so overall results are a weighted average of strata-specific estimates. The use of a dummy variable in the model for district is a more efficient way to take district level effects into account, rather than to create an observation for each district for each week/year/season, which would increase the size of data set 43 times, and require a meta-analysis to combined district-specific estimates. We do present some district-specific results for the large districts, in Additional file 1.

Analysis was conducted using the statistical software Stata 12 (Stata, Inc., Texas, USA) and Excel (Microsoft Office Excel 2007; Microsoft Corporation). A *p*-value < 0.05 was considered as significant. The data were obtained through agreements with the MINSA and SENAMHI. As this is a record based study with no contact with patients, and the data were anonymized, it was not submitted to an Internal Review Board (Ethics Committee).

## Results

During the study period, there were 3,099,438 cases of health center outpatient visits for respiratory diseases of children under 5 years old in Lima province, Peru between 2011 and 2015. Table 1 gives the mean weekly number of visits for the various outcomes by respiratory diseases according age group, as well as the average weekly PM_2.5_ across all years, and the average weekly temperature, and relative humidity values across all years. The population-weighted average PM_2.5_ estimated for Lima province, across all districts and years, was 20.5 μg/m^3^ (SE = 6.3) (Table 1).

**Table 1:** Average weekly values across the study period for outcome and predictor variables, Lima-Peru, 2011–2015

Health center outpatients visits	n	Weekly Mean	SE	Min	Max
ALRI < 2 m	136.173	12.3	15.7	0.0	154.0
ALRI 2-11 m	780.733	70.7	80.0	0.0	543.0
ALRI 1-4a	1737.793	157.3	170.7	0.0	1305.0
PNEU 2-11 m	6.444	0.6	1.2	0.0	28.0
PNEU 1-4a	12.223	1.1	1.9	0.0	24.0
Acute bronchiolitis /Asthma < 2a	242.657	22.0	29.7	0.0	309.0
Acute bronchiolitis /Asthma 2-4a	183.415	16.6	22.2	0.0	223.0
PM_2.5_ (μg/m^3^)		20.5	6.3	9.6	48.6
Relative Humidity (%)		72.9	13.6	40.3	95.2
Temperature		20.8	2.1	15.1	27.5

*SE* standard error, *ALRI* acute lower respiratory infections, *PNEU* pneumonia

**Fig. 1:**
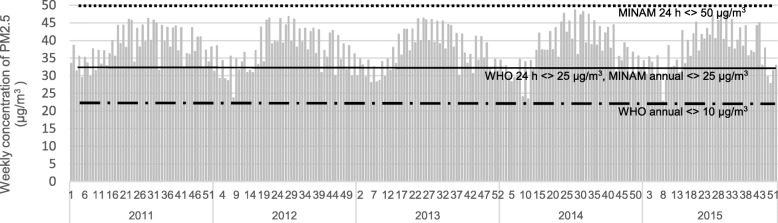
Modeling of PM_2.5_ maximum concentration daily in Lima province, Peru, 2011–2015. = MINAM Air Quality Guidelines 24 h standard (50 μg/m^3^)  — = WHO Air Quality Guidelines 24 h standard (25 μg/m^3^) and MINAM Air Quality Guidelines annual (25 μg/m^3^)  =WHO Air Quality Guidelines annual (10 μg/m^3^) Units on x axis are weekly concentration of PM2.5 (μg/m^3^)

In 20% of the weeks considered, the weekly mean concentrations of PM_2.5_ presented values above the limit daily recommended by the WHO (25 μg/m^3^) and almost 100% presented values above the annual recommended limit (10 μg/m^3^) [11]. In relation to the Peruvian Ministry of the Environmental (MINAM) recommendations, 20% of the weeks considered were above the annual limit (25 μg/m^3^), although none surpassed the daily limit (50 μg/m^3^) (Fig. 1).

Figure 1 shows the distribution daily of concentrations of PM_2.5_, in Lima province, Peru, 2011–2015.

Lag (0) fit better than the other lags according to the AIC for all 3 types of respiratory diseases), and was used in the regressions.

The rate ratios (RR) for outpatient visits for an IQR increase in PM_2.5_ are shown in Table 2. We found a significant association between exposures to PM_2.5_ for all three types of respiratory diseases, and across all age groups. All ALRI increases 6% per IQR, while asthma increases 10% and pneumonia increases 17%.

**Table 2:** Rate ratios for respiratory diseases associated with each interquartile range increase in PM_2.5_ concentration in different age group in Lima province, Peru, 2011–2015^a^

Health center outpatients visits	n	RR	IC 95%		p-value
All ALRI < 5 yr	2654.699	1.06	1.04	1.07	< 0.001
ALRI < 2 mths	136.173	1.06	1.04	1.09	0.01
ALRI 2 mths- < 1 yr	780.733	1.06	1.04	1.07	0.00
ALRI 1- < 5 years	1737.79	1.06	1.05	1.08	< 0.001
All Pneu < 5 yr	18.667	1.17	1.11	1.23	< 0.001
PNEU 2-11 mths	6.444	1.19	1.10	1.28	0.02
PNEU 1- < 5 years	12.223	1.16	1.09	1.22	< 0.001
All Asthma < 5 yr	426.072	1.10	1.08	1.12	< 0.001
Acute bronchiolitis/ Asthma < 2 years	242.657	1.10	1.08	1.13	< 0.001
Acute bronchiolitis/ Asthma 2- < 5 years	183.415	1.11	1.08	1.13	< 0.001

^a^*ALRI* acute lower respiratory infections, *PNEU* pneumonia, *RR* rate ratio, *CI* confidence interval. Negative binomial Model adjusted by temperature, relative humidity, season, year and districts. Average district-level PM_2.5_ during the same week (lag 0) was used as exposure

Table 3 shows analyses by quintile of PM_2.5_. We found consistent monotonic increases in rate ratios with increasing PM_2.5_, with increases of more than 10% in rates of clinic visits in the highest quintile (25.1–48.6 μg/m^3^) in all disease groups.

**Table 3:** Relationship between respiratory diseases with PM_2.5_ quintiles in children under 5 years in Lima-Peru^a^

Health center outpatients visits	Quintile	RR^a^EXP	95% CI	
**ALRI < 2 m**	I (< 15.64)	1		
	II (15.64–17.48)	1.03	0.98	1 .56
	III (17.49–19.71)	1.04**	1.05	1.7
	IV (19.72–25.08)	1.06**	1.09	1.98
	V (25.09–48.62)	1.14**	1.77	3.72
**ALRI 2-11 m**	I (< 15.64)	1		
	II (15.64–17.48)	1.05**	1.2	1.6
	III (17.49–19.71)	1.05**	1.21	1.64
	IV (19.72–25.08)	1.06**	1.26	1.82
	V (25.09–48.62)	1.13**	1.9	3.07
**ALRI 1-4a**	I (< 15.64)	1		
	II (15.64–17.48)	1.04**	1.14	1.49
	III (17.49–19.71)	1.04**	1.15	1.53
	IV (19.72–25.08)	1.07**	1.31	1.88
	V (25.09–48.62)	1.14**	1.98	3.18
**PNEU 2-11 m**	I (< 15.64)	1		
	II (15.64–17.48)	1.00	0.45	2.36
	III (17.49–19.71)	1.01	0.46	2.36
	IV (19.72–25.08)	1.12	0.85	5.74
	V (25.09–48.62)	1.32**	2.14	23.33
**PNEU 1-4a**	I (< 15.64)	1		
	II (15.64–17.48)	1.04	0.74	2.36
	III (17.49–19.71)	1.13**	1.37	4.29
	IV (19.72–25.08)	1.14**	1.26	5.07
	V (25.09–48.62)	1.26**	2.19	12.52
**Acute bronchiolitis /Asthma <2a**	I (< 15.64)	1		
	II (15.64–17.48)	1.07**	1.3	1.98
	III (17.49–19.71)	1.08**	1.41	2.19
	IV (19.72–25.08)	1.07**	1.27	2.19
	V (25.09–48.62)	1.19**	2.4	4.79
**Acute bronchiolitis /Asthma 2-4a**	I (< 15.64)	1		
	II (15.64–17.48)	1.05**	1.11	1.71
	III (17.49–19.71)	1.06**	1.2	1.88
	IV (19.72–25.08)	1.07**	1.25	2.19
	V (25.09–48.62)	1.19	2.44	4.97

^a^Negative binomial Model adjusted by temperature, relative humidity, season, year and districts. Bold values denote statistical significance. ALRI = Acute Lower Respiratory Infections. *PNEU* pneumonia, *RR* rate ratio, *CI* confidence interval. Average district-level PM2.5 during the same week (lag 0) was used as exposure.** = *p* < 0.05

## Discussion

Lima province has 8565.213 inhabitants according to the 2017 National Census [12]. The districts of Lima province have a large childhood population exposed to levels of air pollution well above the recommended annual WHO Air Quality Guidelines for PM_2.5_ (10 μg/m^3^) [11].The association between PM_2.5_ concentrations and health center outpatients visits for respiratory diseases in our study was evident at concentrations which are in the range of the MINAM’s annual permissible level in Lima (25 μg/m^3^) [13, 14]. Our data suggest that it is necessary to take corrective measures to reduce the effects of environmental pollutants, such as PM_2.5_.

We have studied health center outpatient visits, which are generally less severe than visits to emergency rooms, and we have focused on children under 5. Our study found a significant association between an increase of 7.1 μg/m^3^ in PM_2.5_ with increased outpatient visits for respiratory disease, with effects for all types of respiratory disease (ALRI, pneumonia and acute bronchiolitis/asthma).

Most studies in Lima, Peru about contamination by PM_2.5_ are restricted to describing exposure, without any association with disease [2, 8, 14]. On exception is the paper by Tapia et al. (2019), which found that for each interquartile range (IQR) increase in PM_2.5_, respiratory disease, emergency room visits in Lima increased 3% (95% CI: 1.02–1.04%) for those under 18 years [15]. In our study we found a 6% (95% CI: 1.05–1.08%) increase in respiratory clinic visits children under 5 years. It is possible that children under 5 are more susceptible to the effects of air pollution. On the other hand, a recent meta-analysis of 16 time-series studies of hospital admissions in children younger than 5 years, found a respiratory disease increase of 2.7% (95 CI = 0.9–7.7%) per 10 μg / m^3^ increase in PM2. 5 [16], also lower than our estimate.

Our study has some limitations. We don’t know how accurate our local estimates of PM_2.5_ are, although the model [7] had a very good correlation with daily observed levels on the ground (r-square = 0.70). Another possible limitation is that, although data from health center outpatients visits were obtained from an official source (MINAM), they may contain diagnostic errors, and they were available only for epidemiological week, rather than daily. Also we had no data on disease severity, although it is likely that cases were not severe ones, as they did not go to the emergency room.

## Conclusions

Higher emissions of environmental pollutants such as PM_2,5_ could be a trigger for the increase of health center outpatients visits for respiratory diseases (ALRI, pneumonia and asthma), which are themselves risk factors for mortality for children in Lima province, Peru.

## Additional file

**Additional file 1 : Table S1**. Relationship between respiratory diseases with PM_2.5_ quintiles in children under 5 years in Lima-Peru*.

## Data Availability

Data from this study is available upon request.

## Abbreviations

AIC: Akaike information criterion
ALRI: Acute lower respiratory infections
CI: Confidence interval
ICD-10: International Classification of Diseases 10th
IQR: Interquartile range
MINAM: Peruvian Ministry of the Environmental
MINSA: Peru’s Ministry of Health
PNEU: Pneumonia
Q: Quintile
RR: Rate ratio
SE: Standard error
SENAMHI: Peruvian National Service of Meteorology and Hydrology
WHO: World Health Organization
