# Correction to: Association of PM2.5 concentration with health center outpatient visits for respiratory diseases of children under 5 years old in Lima, Peru

**DOI:** 10.1186/s12940-020-0569-0

**Published:** 2020-01-30

**Authors:** Jennifer Estefanía Davila Cordova, Vilma Tapia Aguirre, Vanessa Vasquez Apestegui, Luis Ordoñez Ibarguen, Bryan N. Vu, Kyle Steenland, Gustavo F. Gonzales

**Affiliations:** 1 Faculty of Sciences and Philosophy, and Laboratory of Investigation and Development, Universidad Peruana Cayetano Heredia, Lima, Peru; 2 National Center for Epidemiology, Prevention and Control of Diseases, Minsa, Peru; 3 Department of Environmental Health, Rollins School of Public Health, Emory University, Atlanta, GA 30322 USA

**Correction to: Environ Health (2020) 19:7**

**https://doi.org/10.1186/s12940-020-0564-5**

The original version of this article [1], published on 15 January 2020, contained incorrect name of the co- author. In this Correction the affected part of the article is shown.

Incorrect co-author’s name:

Gustavo F. Gonzales Rengifo

Correct co-author’s name:

Gustavo F. Gonzales

The original article has been corrected.
