# Supplementary material for: Association of PM2.5 concentration with health center outpatient visits for respiratory diseases of children under 5 years old in Lima, Peru
Source: Environ Health. 2020 Jan 15;19:7. doi: 10.1186/s12940-020-0564-5 (PMC6964058; doi:10.1186/s12940-020-0564-5)
Supplement: Supplementary file 1 — Additional file 1 : Table S1. Relationship between respiratory diseases with PM2.5 quintiles in children under 5 years in Lima-Peru*. [file 12940_2020_564_MOESM1_ESM.docx]

Supplementary material 1

Table. Relationship between respiratory diseases with PM_2.5_ quintiles in children under 5 years in Lima-Peru*.

| Health center outpatients visits | District | RR*IQR | 95% CI | |
| --- | --- | --- | --- | --- |
| ALRI <2m | SJL | 1.07** | 1.01 | 1.13 |
|  | San Martin de Porres | 1.01 | 0.87 | 1.16 |
|  | Ate | 1.08 | 0.99 | 1.18 |
|  | Comas | 1.04 | 0.93 | 1.16 |
|  | Villa María del Triunfo | 1.06 | 0.95 | 1.19 |
|  | Villa El Salvador | 1.13** | 0.99 | 1.29 |
|  | San Juan de Miraflores | 1.03 | 0.90 | 1.19 |
|  | Puente Piedra | 1.17** | 1.04 | 1.32 |
|  | Carabayllo | 1.11** | 1.00 | 1.22 |
|  | Los Olivos | 1.10 | 0.94 | 1.29 |
| ALRI 2-11m | SJL | 1.11** | 1.06 | 1.15 |
|  | San Martin de Porres | 1.05 | 0.9877 | 1.2103 |
|  | Ate | 1.09** | 1.02 | 1.16 |
|  | Comas | 1.07 | 0.9911 | 1.1626 |
|  | Villa María del Triunfo | 1.06 | 0.9834 | 1.1499 |
|  | Villa El Salvador | 1.09** | 1.01 | 1.19 |
|  | San Juan de Miraflores | 1.05 | 0.9702 | 1.1291 |
|  | Puente Piedra | 1.09** | 1.01 | 1.19 |
|  | Carabayllo | 1.08** | 1.02 | 1.15 |
|  | Los Olivos | 1.04 | 0.9459 | 1.1485 |
| ALRI 1-4a | SJL | 1.13** | 1.08 | 1.18 |
|  | San Martin de Porres | 1.12** | 1.02 | 1.23 |
|  | Ate | 1.09** | 1.02 | 1.16 |
|  | Comas | 1.08 | 0.9875 | 1.1750 |
|  | Villa María del Triunfo | 1.08** | 1.00 | 1.16 |
|  | Villa El Salvador | 1.13** | 1.05 | 1.22 |
|  | San Juan de Miraflores | 1.06 | 0.9795 | 1.1400 |
|  | Puente Piedra | 1.09** | 1.00 | 1.17 |
|  | Carabayllo | 1.11** | 1.04 | 1.19 |
|  | Los Olivos | 1.10 | 0.998 | 1.219 |
| PNEU 2-11m | SJL | 1.13 | 0.96 | 1.34 |
|  | San Martin de Porres | 1.31 | 0.85 | 2.02 |
|  | Ate | 1.56** | 1.13 | 2.14 |
|  | Comas | 1.03 | 0.71 | 1.49 |
|  | Villa María del Triunfo | 0.80 | 0.57 | 1.11 |
|  | Villa El Salvador | 0.95 | 0.62 | 1.43 |
|  | Puente Piedra | 0.99 | 0.68 | 1.46 |
|  | Carabayllo | 1.68 | 1.07 | 2.64 |
|  | Los Olivos | 1.55 | 0.87 | 2.79 |
|  | Santiago de Surco | 1.28 | 0.5076 | 3.2255 |
| PNEU 1-4a | SJL | 1.15* | 1.01 | 1.30 |
|  | San Martin de Porres | 0.93 | 0.71 | 1.22 |
|  | Ate | 1.10 | 0.86 | 1.40 |
|  | Comas | 1.19 | 0.90 | 1.56 |
|  | Villa María del Triunfo | 0.84 | 0.64 | 1.11 |
|  | Villa El Salvador | 1.34 | 0.93 | 1.94 |
|  | San Juan de Miraflores | 1.04* | 0.75 | 1.43 |
|  | Puente Piedra | 1.30 | 0.93 | 1.80 |
|  | Carabayllo | 0.93 | 0.65 | 1.33 |
|  | Los Olivos | 1.34 | 0.94 | 1.91 |
| Asthma <2a | SJL | 1.13* | 1.06 | 1.20 |
|  | San Martin de Porres | 1.13 | 0.99 | 1.28 |
|  | Ate | 1.19* | 1.10 | 1.29 |
|  | Comas | 1.12* | 1.01 | 1.25 |
|  | Villa Maria del Triunfo | 1.05* | 0.95 | 1.16 |
|  | Villa El Salvador | 1.10 | 0.99 | 1.21 |
|  | San Juan de Miraflores | 1.06 | 0.96 | 1.17 |
|  | Puente Piedra | 1.16* | 1.03 | 1.32 |
|  | Carabayllo | 1.07 | 0.98 | 1.17 |
|  | Los Olivos | 1.23* | 1.07 | 1.42 |
| Asthma 2-4a | SJL | 1.13* | 1.06 | 1.22 |
|  | San Martin de Porres | 1.18* | 1.04 | 1.34 |
|  | Ate | 1.10* | 1.01 | 1.20 |
|  | Comas | 1.06 | 0.98 | 1.15 |
|  | Villa María del Triunfo | 1.15 | 1.03 | 1.28 |
|  | Villa El Salvador | 1.03* | 0.93 | 1.14 |
|  | San Juan de Miraflores | 1.02 | 0.92 | 1.13 |
|  | Puente Piedra | 1.07 | 0.94 | 1.22 |
|  | Carabayllo | 1.11* | 1.01 | 1.22 |
|  | Los Olivos | 1.03 | 0.89 | 1.19 |

*Negative binomial Model adjusted by temperature, relative humidity, season, year and districts. Bold values denote statistical significance. ALRI= Acute Lower Respiratory Infections. PNEU=Pneumonia. RR= Rate ratio, CI= Confidence interval. IQR= Interquartile range (7.1 µg/m^3^). Average district-level PM2.5 during the same week (lag 0) was used as exposure.**=p<0.05.
